# A neutrophil-to-lymphocyte ratio-based prognostic model to predict mortality in patients with HBV-related acute-on-chronic liver failure

**DOI:** 10.1186/s12876-021-02007-w

**Published:** 2021-11-10

**Authors:** Jian Sun, Hongying Guo, Xueping Yu, Haoxiang Zhu, Xueyun Zhang, Jianghua Yang, Jiefei Wang, Zhiping Qian, Zhongliang Shen, Richeng Mao, Jiming Zhang

**Affiliations:** 1 Department of Infectious Diseases, Huashan Hospital, Fudan University, Shanghai, 200040 China; 2 Department of Infectious Diseases, First Affiliated Hospital of Wannan Medical College, Wuhu, 241000 China; 3 Department of Severe Hepatopathy, Shanghai Public Health Clinical Centre, Fudan University, Shanghai, 201508 China; 4 Department of Infectious Diseases, First Hospital of Quanzhou, Affiliated to Fujian Medical University, Quanzhou, 362000 China; 5 Shanghai Key Laboratory of Infectious Diseases and Biosafety Emergency Response, Shanghai, 200040 China

**Keywords:** Acute-on-chronic liver failure, Asian Pacific Society for the Study of the Liver ACLF Research Consortium, Prognosis, Bacterial infection, Neutrophil-to-lymphocyte ratio, Lactate, Cirrhosis

## Abstract

**Background:**

Although the Asian Pacific Association for the Study of the Liver acute-on-chronic liver failure (ACLF) research consortium (AARC) ACLF score is easy to use in patients with hepatitis b virus-related ACLF (HBV-ACLF), serum lactate is not routinely tested in primary hospitals, and its value may be affected by some interference factors. Neutrophil-to-lymphocyte ratio (NLR) is used to assess the status of bacterial infection (BI) or outcomes in patients with various diseases. We developed an NLR-based AARC ACLF score and compared it with the existing model.

**Methods:**

A total of 494 HBV-ACLF patients, enrolled in four tertiary academic hospitals in China with 90-day follow-up, were analysed. Prognostic performance of baseline NLR and lactate were compared between cirrhotic and non-cirrhotic subgroups via the receiver operating curve and Kaplan–Meier analyses. A modified AARC ACLF (mAARC ACLF) score using NLR as a replacement for lactate was developed (n = 290) and validated (n = 204).

**Results:**

There were significantly higher baseline values of NLR in non-survivors, patients with admission BI, and those with higher grades of ACLF compared with the control groups. Compared with lactate, NLR better reflected BI status in the cirrhotic subgroup, and was more significantly correlated with CTP, MELD, MELD-Na, and the AARC score. NLR was an independent predictor of 90-day mortality, and was categorized into three risk grades (< 3.10, 3.10–4.78, and > 4.78) with 90-day cumulative mortalities of 8%, 21.2%, and 77.5% in the derivation cohort, respectively. The mAARC ACLF score, using the three grades of NLR instead of corresponding levels of lactate, was superior to the other four scores in predicting 90-day mortality in the derivation (AUROC 0.906, 95% CI 0.872–0.940, average *P* < 0.001) and validation cohorts (AUROC 0.913, 95% CI 0.876–0.950, average *P* < 0.01), with a considerable performance in predicting 28-day mortality in the two cohorts.

**Conclusions:**

The prognostic value of NLR is superior to that of lactate in predicting short-term mortality risk in cirrhotic and non-cirrhotic patients with HBV-ACLF. NLR can be incorporated into the AARC ACLF scoring system for improving its prognostic accuracy and facilitating the management guidance in patients with HBV-ACLF in primary hospitals.

**Supplementary Information:**

The online version contains supplementary material available at 10.1186/s12876-021-02007-w.

## Background

Acute-on-chronic liver failure (ACLF) is associated with substantial short-term mortality and a high incidence of secondary infection [1–4]. Bacterial infection (BI) is a severe complication and predominates in the aetiology of secondary infection in ACLF [1–4]. Numerous studies have suggested that BI is a vital factor for triggering or aggravating systemic inflammation (SI) in ACLF and contributes to organ failure and poor outcomes in patients with the disease [4–6].

Complex scoring models have been developed to improve short-term survival prediction in patients with ACLF. White blood cell count (WBC), regarded as a critical independent risk factor associated with 28-day mortality in cirrhotic patients with ACLF, has been incorporated in the Chronic Liver Failure Consortium (CLIF-C) ACLF score [7] and the North American Consortium for the Study of End-Stage Liver Disease (NACSELD) ACLF score [8]. In the Asia–Pacific region, hepatitis b virus (HBV) infection is the main aetiology of ACLF, and research has suggested that a significant proportion of ACLF patients are non-cirrhotic or do not have extrahepatic organ failures upon admission [9, 10]. Additionally, most ACLF patients in this region are not treated in the intensive care unit because of objective limitations, impeding data collection for the two aforementioned scores.

Four frequently-used indexes (including total bilirubin [TB], hepatic encephalopathy [HE] grade, international normalized ratio [INR], and creatinine) and one metabolic index (serum lactate) are used for severity prediction in cirrhotic or non-cirrhotic patients with ACLF, per the Asian Pacific Society for the Study of the Liver (APSAL) ACLF Research Consortium (AARC) ACLF score [11]. Compared with the CLIF-C or NACSELD ACLF score, the AARC ACLF score is easier to calculate and suits more patients with HBV-ACLF in the Asia–Pacific region [10–12]. However, the value of serum lactate may be affected by several interference factors, like the source of the blood sample and the cirrhotic status of a patient. Additionally, serum lactate is not a routine testing item in primary hospitals in this region, impeding the promotion of the lactate-based AARC ACLF score.

Increased neutrophil/lymphocyte ratio (NLR), a physiological response of circulating leucocytes to inflammatory stress, serves as a useful biomarker to reflect the status of BI or SI in various inflammatory conditions [13, 14]. It is used for survival prediction before or after a liver transplant [15–17]. Recent studies have suggested that NLR is a novel biomarker for predicting short-term mortality particularly 90-day mortality, not only in patients with non-HBV ACLF [18–20] but also in those with HBV-ACLF [21–23]. However, it is unknown whether incorporating NLR into the existing AARC ACLF score can improve its prognostic value. Therefore, we designed this multi-centre study to develop an NLR-based AARC ACLF score to predict short-term mortality risk in patients with HBV-ACLF.

## Methods

### Study design

This retrospective study evaluated consecutive admissions of HBV-ACLF patients to four academic centres (Huashan Hospital, Shanghai; Shanghai Public Health Clinical Centre, Shanghai; First Affiliated Hospital of Wannan Medical College, Wuhu; and First hospital of Quanzhou affiliated to Fujian Medical University, Quanzhou) in China from January 2013 to December 2019. Two investigators at each centre were responsible for reviewing the patient charts to (a) diagnose and grade ACLF according to the APSAL criteria; (b) confirm the presence of cirrhosis and BI; and (c) assess survival. Any discrepancy between the two investigators was adjudicated by a senior physician.

### Patient selection-derivation and validation cohorts

**Fig. 1:**
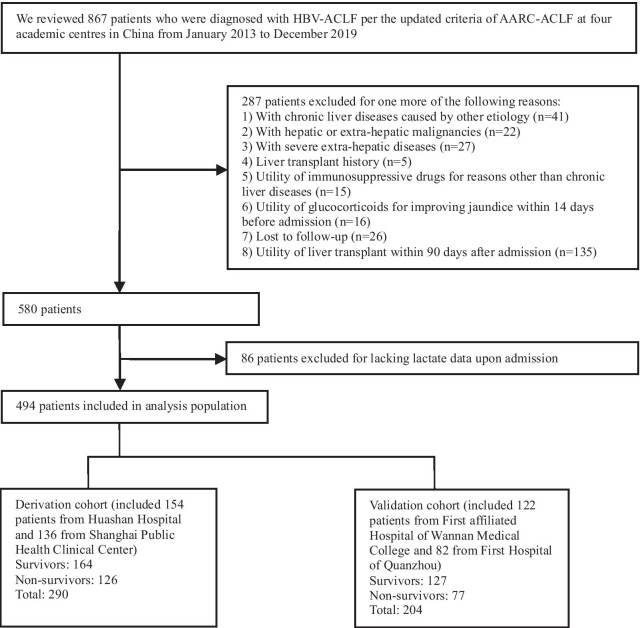
Flow chart of the study design. ACLF was diagnosed and graded according to the criteria of the APASL ACLF Research Consortium (AARC). Eighty-six patients (21 patients from the derivation cohort and 65 from the validation cohort) had no baseline data of lactate and were excluded. Survival status (or hospital mortality, whichever occurred sooner) was identified using follow-up for 90 days after admission. Abbreviations: ACLF, acute-on-chronic liver failure; HBV-ACLF, hepatitis b related ACLF; AARC, Asian Pacific Society for the Study of the Liver ACLF research consortium

A total of 867 HBV-ACLF patients were retrospectively screened at the four enrolled centres from 2013 to 2019. Of these, 494 patients were transplant-free with complete data for ascertainment of the study outcome and were finally included. The criteria of inclusion and exclusion are summarized in Fig. 1. Two hundred and ninety patients from Huashan Hospital and Shanghai Public Health Clinical Centre were evaluated as the derivation cohort, and 204 patients from the other two centres were evaluated as the validation cohort. All patients received standard medical treatment, and antibiotic therapy was managed in patients with proven or suspected BI. Per the national guideline for non-bioartificial liver support systems in the treatment of liver failure, artificial liver support was utilized after comprehensive evaluation of the clinical state of a patient with informed consent [24].

### Data collection and follow-up

Collected data included patient demographics, medical history, clinical parameters, and data of laboratory, radiologic, and microbiologic tests. Baseline data were defined as the poorest result/score obtained in the first 24-h period during hospitalization. Calculated severity scores were as follows: Child-Turcotte-Pugh (CTP) [25], model of end-stage liver disease (MELD) [26], MELD-Na [27], and AARC ACLF score [11]. All patients were followed up for 90 days with respect to their clinical outcome (or hospital mortality, whichever came sooner). Leucocytes were quantified using flow-cytometry-based cell counters in the four enrolled centres from 2013 to 2019. This approach can reduce the discrepancy in the value of NLR and increase subsequent prognostic accuracy [19].

### Definition

ACLF was diagnosed and graded according to the updated criteria of AARC-ACLF, irrespective of the presence of cirrhosis or extrahepatic organ failures [10]. Diagnosis of cirrhosis was mainly based on clinical presentation, biochemical, and radiological evidence (e.g., shrinkage or superficial changes of the liver, portal hypertension, ascites, and splenomegaly); some patients undertook liver biopsy or endoscopy or FibroScan previously, and their reports were taken into consideration either. BI was diagnosed based on a comprehensive evaluation that included clinical symptoms, laboratory and/or imaging findings, and positive results of microbiological tests. Admission BI, categorized as community-acquired or healthcare-associated infections per the conventional criteria [28], was diagnosed in patients within 48 h of admission to any of the four enrolled centres.

### Statistical analyses

Continuous variables with normal distributions were described using the mean ± SD and compared using Student’s *t* test. Continuous variables with skewed distributions, described using median with the interquartile range (IQR), were compared using the Mann‐Whitney U test for the comparison of two variables, and the Kruskal–Wallis test was used for the comparison of more than two groups. Categorical variables, expressed as percentages (frequencies), were compared using the Chi**-**square test followed by Fisher’s exact test, as appropriate. Cut-off values of NLR and the mAARC ACLF score for 90-day mortality prediction were calculated using the receiver operating characteristic (ROC) analysis. The area under ROC (AUROC) comparisons were performed via the Hanley & McNeil method. Survival analyses were performed for 90 days by the Kaplan**–**Meier (K**–**M) method. Cox regression models were used for univariate and multivariate analysis of 90-day mortality predictors. Two-tailed *P* value < 0.05 was considered as statistically significant. Statistical analyses were performed using SPSS v22.0 (IBM Inc) and MedCalc v20.008 (MedCalc Software).

## Results

### Baseline characteristics

Table 1 summarises the baseline characteristics of patients in the derivation and validation cohorts. The derivation cohort included 290 patients; the median age was 44 (37–54) years; 86.9% were men; and 232 were admitted or transferred to a liver intensive therapy unit. Patients were graded per the AARC ACLF score, as follows: < 8 in 70 (24.1%) patients; 8–10 in 139 (47.9%); and > 10 in 81 (27.9%). Compared with survivors, non-survivors had a higher proportion of old age, cirrhosis, ascites, poor hepatic/extra-hepatic performance, AARC ACLF score > 10, and history of artificial liver support or antibiotic management (Additional file 1: Table S1). Admission BI was diagnosed in 123 (42.4%) patients, and was observed more in non-survivors (Additional file 1: Table S1). Admission BI most frequently manifested as pneumonia (26.2%), followed by spontaneous bacterial peritonitis (SBP) (17.9%) (Additional file 1: Table S2).

**Table 1:** Baseline characteristics of the study population in derivation and validation cohorts

Variable	Total(n = 494)	Derivationcohort (n = 290)	Validationcohort (n = 204)	*P*value
Age (years)	45 (37–55)	44 (37–54)	46 (38–58)	0.172
Male,% (n/N)	85.2 (421/494)	86.9 (252/290)	82.8 (169/204)	0.211
HBeAg positive,% (n/N)	43.3 (214/494)	44.5 (129/290)	41.7 (85/204)	0.534
HBV DNA,% (n/N)				
≤ 200 IU/ml	30.0 (148/494)	35.2 (102/290)	22.5 (46/204)	< 0.001
200–2 × 10 ^4^ IU/ml	36.2 (179/494)	39.3 (114/290)	31.8 (65/204)	
≥ 2 × 10 ^4^ IU/ml	33.8 (167/494)	25.5 (74/290)	45.7 (93/204)	
Cirrhosis,% (n/N)	77.1 (381/494)	76.9 (223/290)	77.5 (158/204)	0.885
Ascites,% (n/N)	66.2 (327/494)	65.2 (189/290)	67.6 (138/204)	0.567
Bacterial infection,% (n/N)	38.5 (190/494)	42.4 (123/290)	32.8 (67/204)	0.031
Laboratory test				
WBC (10^9^ /L)	6.3 (4.7–9.1)	6.8 (5.0–9.8)	5.9 (4.4–8.1)	0.005
NLR	4.3 (2.5–7.9)	4.6 (2.5–7.9)	4.1 (2.4–7.7)	0.272
Platelets (10^9^ /L)	90 (59–129)	90.5 (59–128)	90 (58.5–131)	0.871
TB (mg/dL)	323 (205–467)	333 (209–467)	310 (201–467)	0.573
Albumin (g/L)	32.4 ± 5.3	32.8 ± 5.2	31.7 ± 5.4	0.012
Creatinine (µmol/L)	66 (54–81)	66 (56–81)	65 (53–82)	0.524
Sodium (mmol/L)	137 (133–139)	136 (132–139)	137 (133–139)	0.079
Glucose (mmol/L)	5.7 (4.4–7.8)	5.7 (4.4–7.5)	5.7 (4.1–8.5)	0.583
Lactate (mmol/L)	1.9 (1.3–2.6)	2.0 (1.3–2.7)	1.8 (1.3–2.4)	0.238
INR	1.9 (1.6–2.5)	2.1 (1.7–2.8)	1.8 (1.5–2.3)	< 0.001
HE grade	0 (0–1)	0 (0–1)	0 (0–1)	0.360
Severity score				
CTP score	11 (10–12)	11 (10–12)	11 (10–12)	0.341
MELD score	22 (18–27)	23 (19–28)	21 (18–25)	0.014
MELD-Na score	23 (19–30)	24 (19–32)	22 (19–28)	0.014
AARC ACLF score	9 (7–10)	9 (8–11)	8.5 (7–10)	0.002
5–7	28.7 (142/494)	24.1 (70/290)	35.3 (72/204)	0.010
8–10	47.0 (232/494)	47.9 (139/290)	45.6 (93/204)	
11–15	24.3 (120/494)	27.9 (81/290)	19.1 (39/204)	
Antiviral history,% (n/N)				
Naïve	71.3 (352/494)	69.3 (201/290)	74.0 (151/204)	0.255
Non–naïve	28.7 (142/494)	30.7 (89/290)	26.0 (53/204)	0.255
Poor adherence	76.8 (109/142)	78.7 (70/89)	73.6 (39/53)	0.489
Antibiotic history,% (n/N)	36.4 (180/494)	41.0 (119/290)	29.9 (61/204)	0.011
Artificial liver support history,% (n/N)	19.2 (95/494)	21.0 (61/290)	16.7 (34/204)	0.225

*HBeAg* hepatitis b envelope antigen, *HBV* hepatitis b virus, *WBC* white blood cell count, *NLR* neutrophil/lymphocyte ratio, *TB* total bilirubin, *INR* international normalized ratio, *HE* hepatic encephalopathy, *CTP* Child-Turcotte-Pugh, *MELD* model for end-stage liver disease, *ACLF* acute-on-chronic liver failure, *AARC* Asian Pacific Society for the Study of the Liver ACLF research consortium

Continuous variables with normal distributions were described using mean ± SD. Continuous variables with skewed distributions were described using median with interquartile range (IQR) and compared using the Mann–Whitney U test. Categorical variables were expressed as percentages (frequencies) and compared using the Chi‐square test followed by Fisher’s exact test, as appropriate

### Relation among BI status, ACLF grade, and short-term mortality risk

**Fig. 2:**
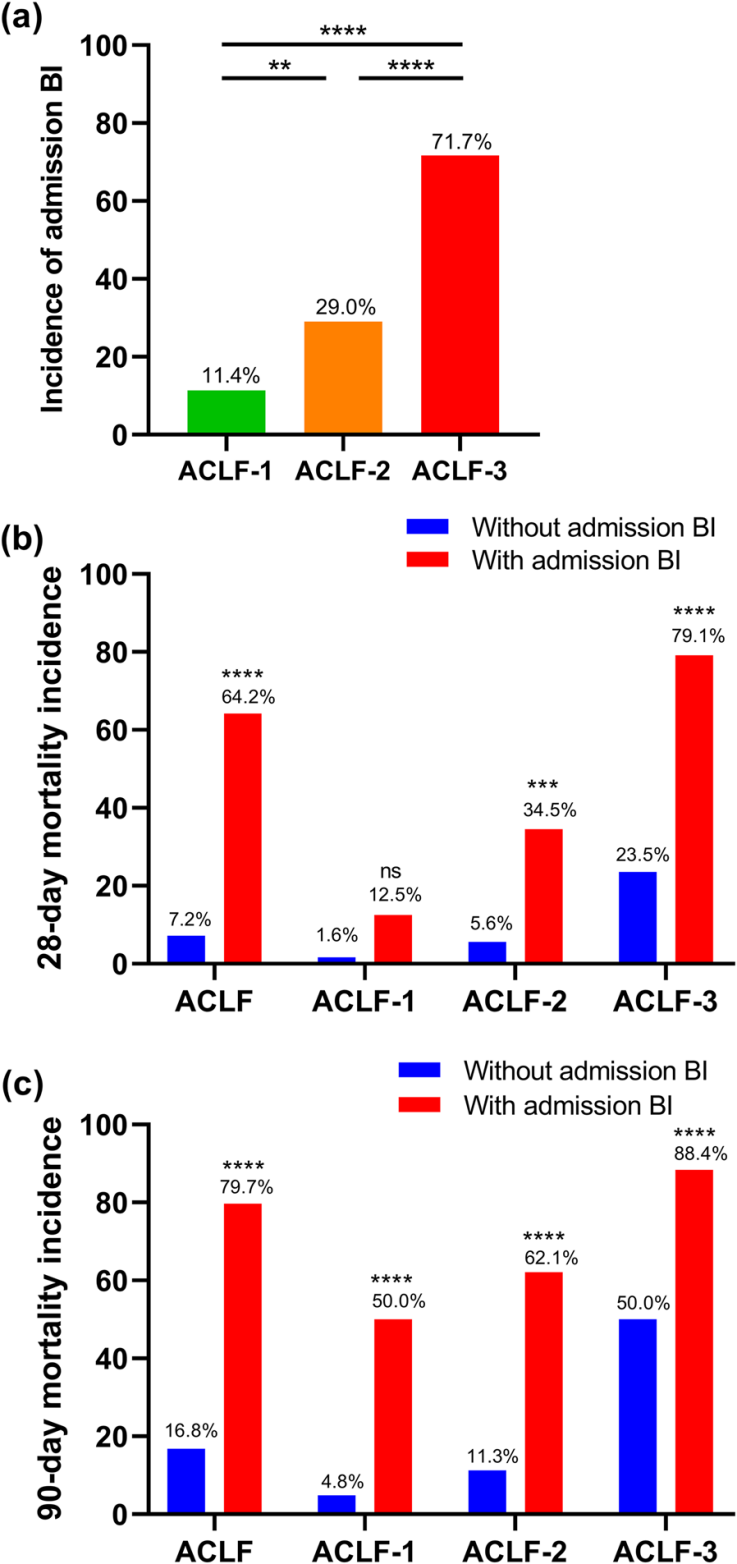
Correlation among BI status, ACLF grade, and short-term mortality was analysed in 290 patients with HBV-ACLF in the derivation cohort. **a** Comparison of admission BI incidence among three patient subgroups (ACLF-1, n = 70; ACLF-2, n = 139; ACLF-3, n = 81); **b** The 28-day mortality incidence was compared between patients with (n = 123) or without admission BI (n = 167), and was further compared within the ACLF-1 (with BI, n = 8; without BI, n = 62), ACLF-2 (with BI, n = 29; without BI, n = 71), and ACLF-3 (with BI, n = 86; without BI, n = 34) subgroups. **c** The 90-day mortality incidence was compared likewise. Categorical data are expressed as percentages (frequencies) and analysed using the Chi‐square test followed by Fisher’s exact test, as appropriate. *P < 0.05, **P < 0.01, ***P < 0.001, ****P < 0.0001. ACLF was graded per the AARC ACLF score (5–7; 8–10; 11–15). Abbreviations: BI, bacterial infection; ACLF, acute-on-chronic liver failure; HBV-ACLF, hepatitis b related ACLF; AARC, Asian Pacific Society for the Study of the Liver ACLF research consortium

Figure 2 summarises the correlation among BI status, AARC ACLF grade, and 28/90-day mortality risk in 290 patients evaluated in the derivation cohort. Admission BI incidence had a positive correlation with the AARC ACLF grade, particularly for those diagnosed with ACLF grade 3 (Fig. 2a). Overall, there was a significantly higher incidence of 28/90-day mortality in ACLF patients admitted with BI, particularly in those with higher ACLF grades (Fig. 2b, c). However, there was no statistical difference in the 28-day mortality incidence between patients with and without admission BI (Fig. 2b).

### Comparisons for performance of NLR and lactate among different patient subgroups

**Fig. 3:**
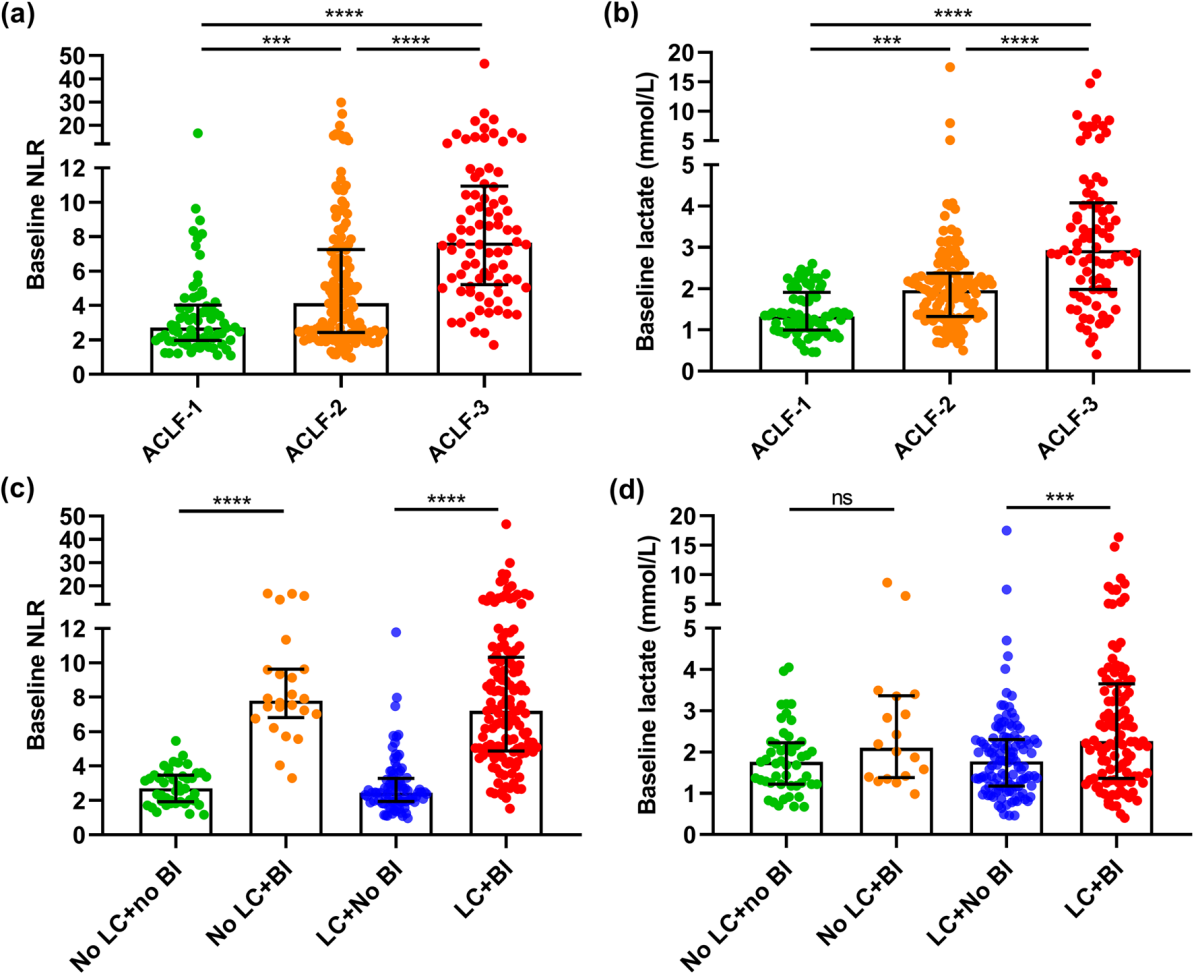
Performance of NLR and lactate in assessment of ACLF progression and BI status was compared in 290 patients with HBV-ACLF in the derivation cohort. **a** Comparison of baseline values of NLR among three patient subgroups (ACLF-1, n = 70; ACLF-2, n = 139; ACLF-3, n = 81); **b** Comparison of baseline values of lactate among the above-mentioned three subgroups; **c** Comparison of baseline values of NLR among four patient subgroups (without LC or BI, n = 43; without LC and with BI, n = 24; with LC and without BI, n = 86; with LC and with BI, n = 137); **d** Comparison of baseline values of lactate among the above-mentioned four patient subgroups. Continuous data are expressed using median (IQR) and compared using the Kruskal–Wallis test. *P < 0.05, **P < 0.01, ***P < 0.001, ****P < 0.0001. ACLF was graded per the AARC ACLF score (5–7; 8–10; 11–15). Abbreviations: NLR, neutrophil-to-lymphocyte ratio; LC, liver cirrhosis; BI, bacterial infection; ACLF, acute-on-chronic liver failure; HBV-ACLF, hepatitis b related ACLF; AARC, Asian Pacific Society for the Study of the Liver ACLF research consortium

In the derivation cohort, non-survivors had higher values of NLR and lactate compared with survivors (Additional file 1: Table S1). Performance of the two variables was further compared among different patient subgroups and summarized in Fig. 3. Overall, there were significantly higher values of NLR (Fig. 3a) and lactate (Fig. 3b) in patients with higher ACLF grades than those in the control subgroups. Patients with admission BI had significantly higher NLR values compared with those without admission BI, with no discrepancy in the cirrhotic or non-cirrhotic subgroup (Fig. 3c). Conversely, the positive correlation between BI and lactate was only observed in the cirrhotic subgroup (Fig. 3d).

### Comparisons for performance of baseline NLR and lactate in disease severity and short-term mortality

**Fig. 4:**
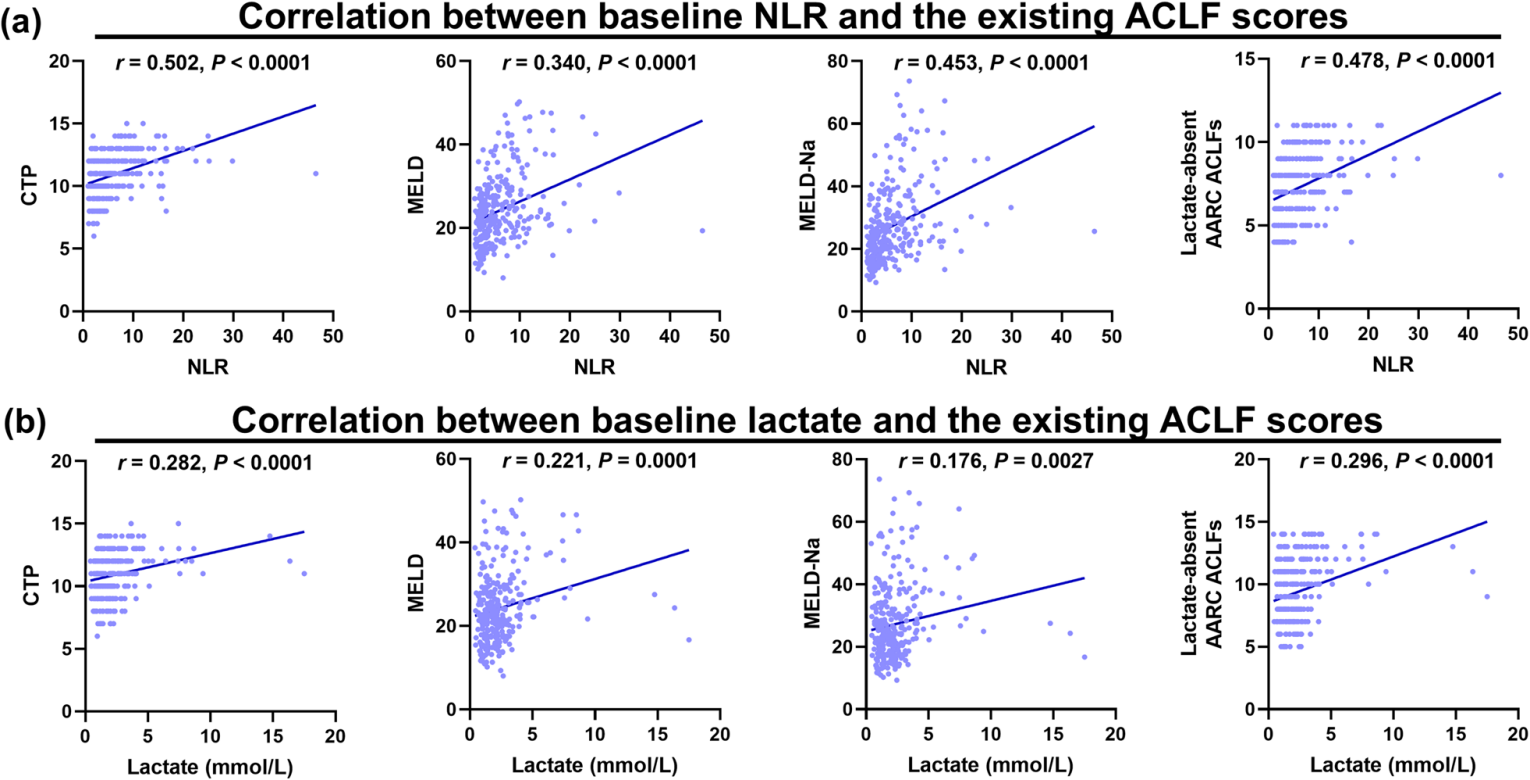
The correlation between accepted severity scores and NLR or lactate were compared using Spearman analysis in 290 patients in the derivation cohort, and was stratified for performance of baseline NLR using the CTP score (**a**), the MELD score (**b**), the MELD-Na score (**c**), and the lactate-absent AARC ACLFs (**d**); for performance of baseline lactate using the CTP score (**e**), MELD score (**f**), MELD-Na score (**g**), and the lactate-absent AARC ACLFs (**h**). The lactate-absent AARC ACLFs was calculated based on the AARC ACLFs and the item of lactate was excluded for an objective result. Abbreviations: NLR, neutrophil-to-lymphocyte ratio; ACLF, acute-on-chronic liver failure; CTP, Child-Turcotte-Pugh; MELD, model of end-stage liver disease; AARC ACLFs, Asian Pacific Society for the Study of the Liver ACLF research consortium ACLF score

**Fig. 5:**
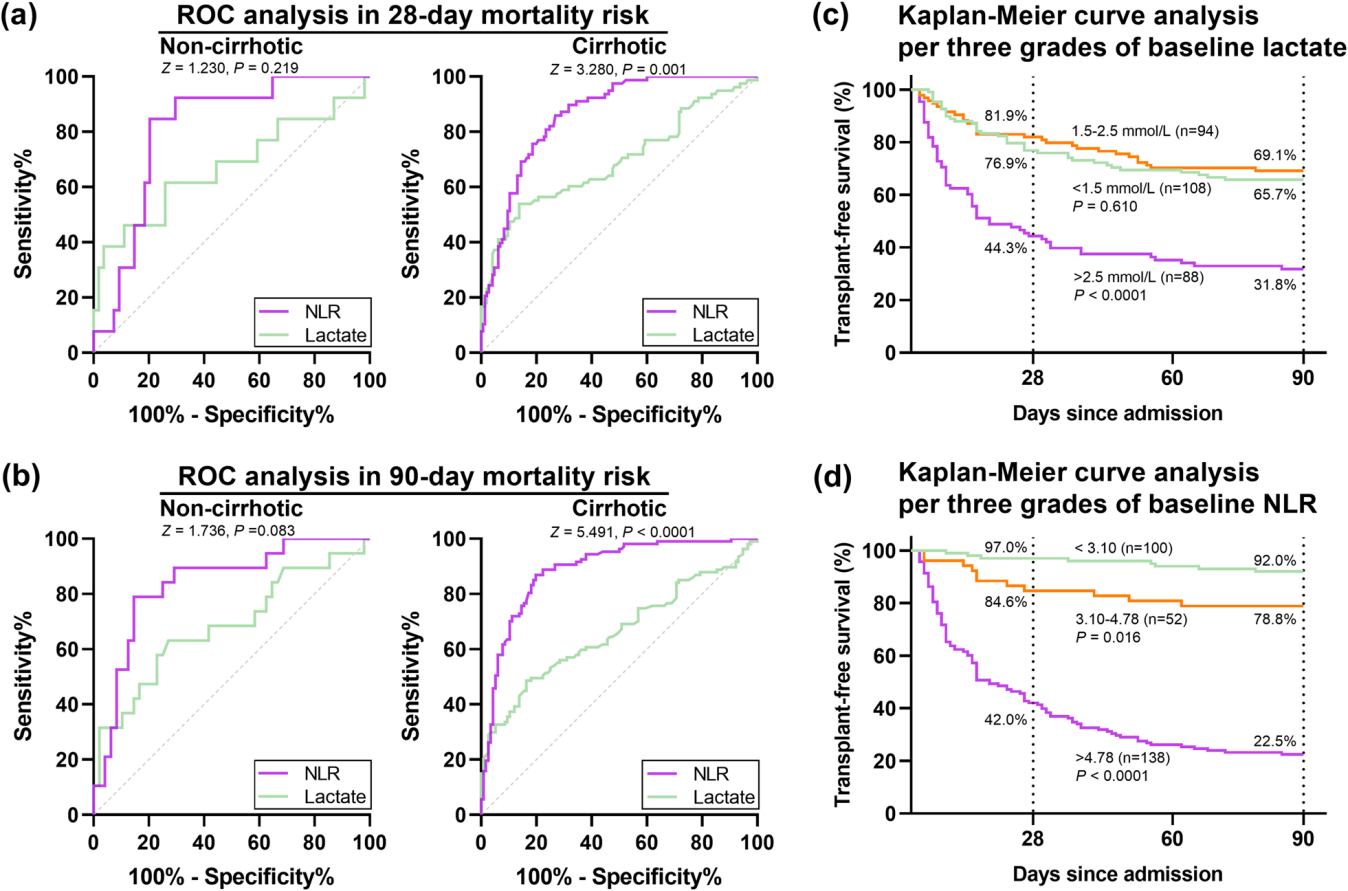
Performance of NLR and lactate in predicting short-term mortality risk was compared in 290 patients with HBV-ACLF in the derivation cohort. **a** Receiver operating characteristics (ROC) curve analyses for 28-day mortality risk in non-cirrhotic (n = 67) or cirrhotic subgroup (n = 223); **b** ROC analyses for 90-day mortality risk in the above-mentioned two subgroups; **c** Kaplan–Meier analyses for 90-day survival based on three risk levels of baseline lactate; **d** KM analyses for 90-day survival based on three risk levels of baseline NLR. Lactate was graded into three risk levels (< 1.5 mmol/L, 1.5–2.5 mmol/L, and > 2.5 mmol/L) according to the AARC ACLF score. NLR was divided into three risk levels (< 3.10, 3.10–4.78, and > 4.78) based on the upper limit of normal and the optimal cut-off value evaluated in the sensitivity analyses in the derivation cohort. The Hanley & McNeil method was used for AUROC comparisons. Abbreviations: NLR, neutrophil-to-lymphocyte ratio; ACLF, acute-on-chronic liver failure; HBV-ACLF, hepatitis b related ACLF; AARC, Asian Pacific Society for the Study of the Liver ACLF research consortium

Spearman analyses showed that NLR correlated well with the CTP score, the MELD score, the MELD-Na score, and the lactate-absent AARC ACLF score (Fig. 4a), and lactate had weaker correlation with the above-mentioned four scores compared with NLR (Fig. 4b). Compared with lactate, NLR had more excellent performance in predicting 28-day mortality (AUROC 0.713, 95% CI 0.649–0.771; AUROC 0.854, 95% CI 0.802–0.898) and 90-day mortality (AUROC 0.670, 95% CI 0.605–0.731; AUROC 0.887, 95% CI 0.837–0.926) in the cirrhotic subgroup, whereas there were no statistical difference between lactate and NLR in predicting 90-day mortality (AUROC 0.684, 95% CI 0.559–0.792; AUROC 0.838, 95% CI 0.727–0.916) and particularly 28-day mortality (AUROC 0.672, 95% CI 0.457–0.782; AUROC 0.809, 95% CI 0.695–0.895) in the non-cirrhotic subgroup (Fig. 5a, b).

The optimal cut-off value for baseline NLR in predicting 90-day mortality risk was 4.78. Kaplan–Meier survival analyses showed that patients with a low value of lactate (< 1.5 mmol/L) and those with a medium value (1.5–2.5 mmol/L) had equal short-term survival rate (Fig. 5c). Conversely, the performance of NLR was excellent among patient subgroups divided via three risk levels of baseline NLR (< 3.10, 3.10–4.78, and > 4.78); NLR > 4.78 was associated with a 58.0% 28-day mortality risk and 77.5% 90-day mortality risk (Fig. 5d).

### Predictors of 90-day mortality risk in patients with HBV-ACLF

Baseline data obtained from 290 patients in the derivation cohort were subjected to Cox regression analyses for identifying 90-day mortality predictors. Univariate and multivariate analyses were conducted by common variables (model 1), and three risk grades of NLR and five AARC-ACLF-related variables (model 2). NLR, HE, INR, and creatinine were consistently predictors of 90-day mortality as assessed using multivariate analyses controlled by the above-mentioned two Cox regression models, whereas lactate was not independently associated with 90-day mortality using multivariate analyses controlled by the model 2 (Table 2). The modified AARC ACLF score (mAARC ACLF score) was generated by using three grades of NLR (< 3.10, 3.10–4.78, and > 4.78) as a replacement of the corresponding levels of lactate (< 1.5 mmol/L, 1.5–2.5 mmol/L, and > 2.5 mmol/L).

**Table 2:** Cox regression analysis for predictors of 90-day mortality in 290 patients with HBV-ACLF in the derivation cohort

	Univariate			Multivariate		
	HR	95% CI	*P*	HR	OR (95% CI)	*P*
Parameter (model 1)						
Age (years)	1.030	1.016–1.045	< 0.001			
Ascites (Y:N)	3.882	2.380–6.330	< 0.001			
HE (Y:N)	9.931	6.445–15.302	< 0.001	5.129	3.209–8.199	< 0.001
WBC (10^9^ /L)	1.150	1.114–1.187	< 0.001			
NLR	1.107	1.087–1.129	< 0.001	1.067	1.038–1.096	< 0.001
Platelets (10^9^ /L)	0.996	0.992–0.999	0.025			
INR	1.925	1.728–2.145	< 0.001	1.487	1.310–1.687	< 0.001
TB (mg/dL)	1.050	1.034–1.067	< 0.001			
Albumin (g/L)	0.970	0.935–1.005	0.096			
Creatinine (mg/dL)	1.642	1.405–1.919	< 0.001	1.269	1.043–1.545	0.017
Sodium (mmol/L)	0.911	0.884–0.939	0.001			
Lactate (mmol/L)	1.282	1.214–1.354	< 0.001	1.192	1.107–1.283	< 0.001
Parameter (model 2)						
NLR grade	4.725	3.364–6.636	< 0.001	3.506	2.481–4.955	< 0.001
INR grade	3.763	2.854–4.963	< 0.001	2.400	1.803–3.193	< 0.001
TB grade	1.745	1.393–2.185	< 0.001			
Creatinine grade	2.166	1.547–3.032	< 0.001	1.727	1.278–2.335	< 0.001
Lactate grade	1.857	1.479–2.332	< 0.001			
HE grade	4.349	3.442–5.496	< 0.001	2.366	1.793–3.121	< 0.001

*HE* hepatic encephalopathy, *WBC* white blood cell count, *NLR* neutrophil/lymphocyte ratio, *INR* international normalized ratio, *TB* total bilirubin, *ACLF* acute-on-chronic liver failure, *HBV-ACLF* hepatitis b related ACLF, *AARC* Asian Pacific Society for the Study of the Liver ACLF research consortium

Model 1 was adjusted for common variables. Model 2 was adjusted for three risk grades of NLR and five AARC-ACLF-related variables (include INR, TB, creatinine, lactate, and HE). Three risk grades of INR (< 1.8, 1.8–2.5, and > 2.5), TB (< 15 mg/dL, 15–25 mg/dL, and > 25 mg/dL), creatinine (< 0.7 mg/dL, 0.7–1.5 mg/dL, and > 1.5 mg/dL), lactate (< 1.5 mmol/L, 1.5–2.5 mmol/L, and > 2.5 mmol/L), and HE (< 3.10, 3.10–4.78, and > 4.78) were divided per the AARC ACLF scoring system. The corresponding three grades of NLR (< 3.10, 3.10–4.78, and > 4.78) was divided per the upper limit of normal and the optimal cut-off value evaluated in the sensitivity analyses in this cohort

### Predictive performance of the mAARC ACLF score

**Fig. 6:**
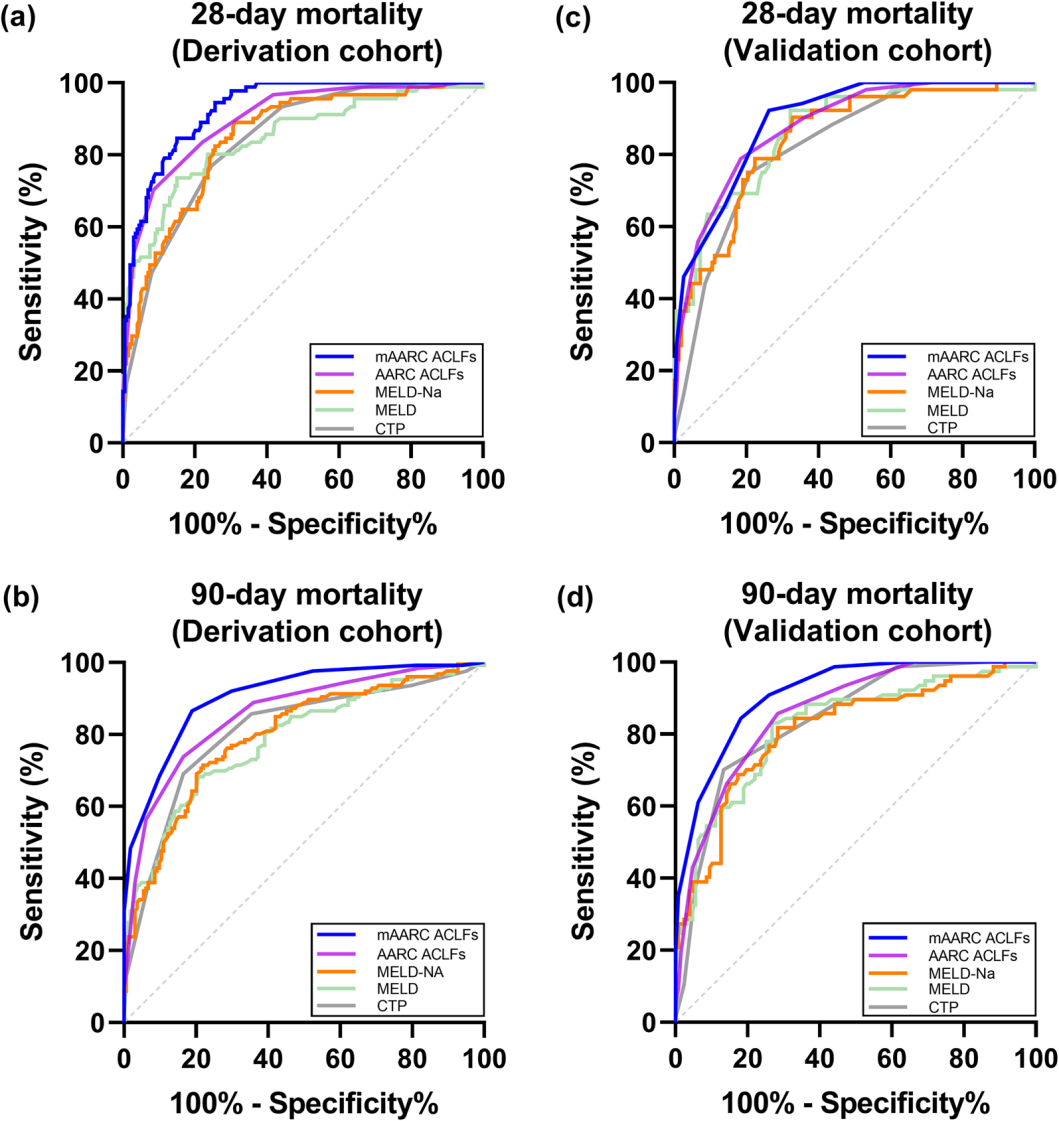
Prognostic performance of the accepted ACLF scores and the mAARC ACLFs was compared using the area under the receiver operating curve (AUROC), and was stratified for their performance in 290 patients in the derivation cohort by **a** comparison of 28-day mortality and **b** comparison of 90-day mortality; for their performance in 204 patients in the validation cohort by **c** comparison of 28-day mortality and **d** comparison of 90-day mortality. The mAARC ACLFs was calculated using three risk levels of baseline NLR (< 3.10, 3.10–4.78, and > 4.78) instead of corresponding levels of lactate (< 1.5 mmol/L, 1.5–2.5 mmol/L, and > 2.5 mmol/L). The Hanley & McNeil method was used for AUROC comparisons. Abbreviations: NLR, neutrophil-to-lymphocyte ratio; ACLF, acute-on-chronic liver failure; CTP, Child-Turcotte-Pugh; MELD, model of end-stage liver disease; AARC ACLFs, Asian Pacific Society for the Study of the Liver ACLF research consortium ACLF score; mAARC ACLFs, modified AARC ACLFs

**Fig. 7:**
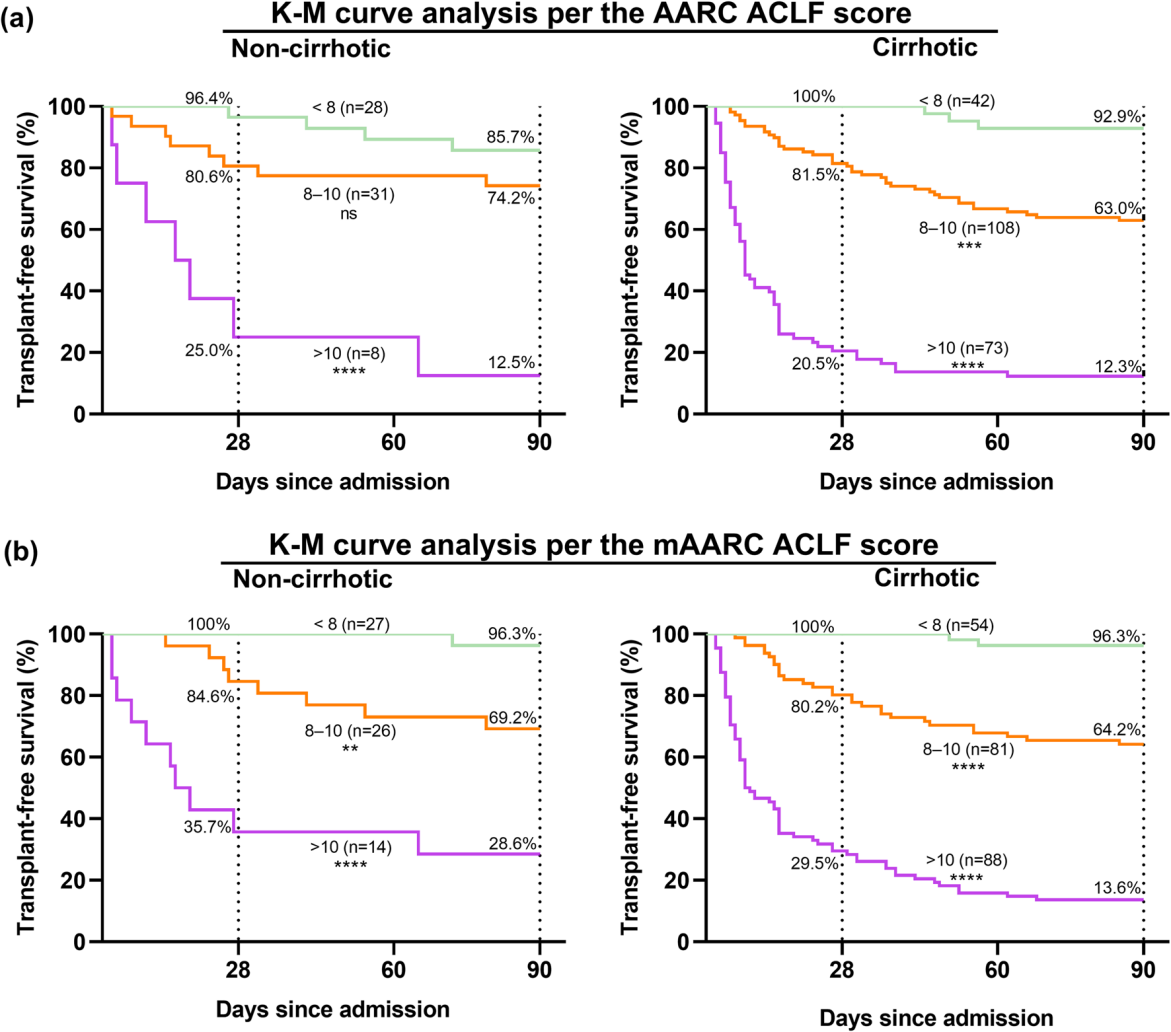
Prognostic performance of the AARC ACLF score and the mAARC ACLF score was compared in the derivation cohort using Kaplan–Meier (K–M) analysis, and was stratified for the performance of the AARC ACLF score in the non-cirrhotic (n = 67) or cirrhotic subgroup (n = 223) using (**a**); for the performance of the mAARC ACLF score in above-mentioned two subgroups using (**b**). The mAARC ACLF score was calculated using three risk levels of baseline NLR (< 3.10, 3.10–4.78, and > 4.78) instead of corresponding levels of lactate (< 1.5 mmol/L, 1.5–2.5 mmol/L, and > 2.5 mmol/L). Abbreviations: ACLF, acute-on-chronic liver failure; AARC, Asian Pacific Society for the Study of the Liver ACLF research consortium

Overall, the performance of the mAARC ACLF score for predicting 28-day mortality (AUROC 0.927, 95% CI 0.891–0.954, average *P* < 0.05) and particularly 90-day mortality (AUROC 0.906, 95% CI 0.866–0.937, average *P* < 0.001) was superior to that of the CTP score, the MELD score, the MELD-Na score, and the AARC ACLF score (Fig. 6a, b). Kaplan–Meier survival analyses showed that there was equal short-term survival rate between non-cirrhotic patients with low or medium AARC ACLF score (Fig. 7a), and dividing ACLF grades via corresponding values of the mAARC ACLF score had excellent performance in the cirrhotic or non-cirrhotic subgroup (Fig. 7b).

Prognostic interest of the mAARC ACLF score was further compared with the above-mentioned four scores in the cirrhotic or non-cirrhotic subgroup. For predicting 28-day mortality risk, performance of the mAARC ACLF score was not inferior to that of the other four scores in the non-cirrhotic subgroup (AUROC 0.916, 95% CI 0.822–0.970, average *P* > 0.05); its performance (AUROC 0.926, 95% CI 0.884–0.957, average *P* < 0.01) was superior to that of CTP, MELD, and MELD-Na except the AARC ACLF score (AUROC 0.903, 95% CI 0.856–0.939, *P* = 0.079) in the cirrhotic subgroup (Additional file 2: Fig. S1a). For predicting 90-day mortality risk, prognostic value of the mAARC ACLF score was superior to the other four scores, not only in the cirrhotic subgroup (AUROC 0.916, 95% CI 0.872–0.949, average *P* < 0.001) but also in the non-cirrhotic subgroup (AUROC 0.850, 95% CI 0.741–0.925, average *P* < 0.05) (Additional file 2: Fig. S1b).

### Validation cohort

Of 269 patients meeting the enrollment criteria in the validation cohort, few were managed in a liver intensive therapy unit, 65 of 269 (24.2%) did not have baseline tests of serum lactate, and 204 patients were finally evaluated (Fig. 1). Compared with the derivation cohort, this cohort had a lower incidence of BI, lower severity scores except the CTP score, and a smaller patient proportion with the AARC ACLF score > 10 or antibiotic history up admission; there was no significant discrepancy in other patient demographics between the two cohorts (Table 1). Admission BI, most frequently manifested as SBP (Additional file 1: Table S2), was observed more in non-survivors compared with that in survivors (Additional file 1: Table S3). The baseline value of NLR was higher in non-survivors than that in survivors (Additional file 1: Table S3), and correlated well with the above-mentioned four scores (average *P* < 0.001).

ROC analysis showed that performance of NLR was superior to that of lactate in predicting 28-day mortality (AUROC 0.876, 95% CI 0.822–0.917; AUROC 0.681, 95% CI 0.612–0.744) (Additional file 3: Fig. S2a) and 90-day mortality (AUROC 0.893, 95% CI 0.842–0.932; AUROC 0.610, 95% CI 0.539–0.677) (Additional file 3: Fig. S2b). Kaplan–Meier analyses showed that in patients with a low or median value of risk index, grading patients via three risk levels of NLR had significantly greater interest compared with using the corresponding levels of lactate (Additional file 3: Fig. S2c, Additional file 3: Fig. S2d).

Despite being a less sick cohort, the performance of the mAARC ACLF score (AUROC 0.895, 95% CI 0.845–0.933, average *P* > 0.05) was not inferior to that of the above-mentioned four scores in 28-day mortality prediction (Fig. 6c), and its performance (AUROC 0.913, 95% CI 0.866–0.948, average *P* < 0.01) was superior to them in 90-day mortality prediction (Fig. 6d). There was a linear correlation between increasing short-term mortality risk and elevating ACLF grades, per the AARC ACLF score (Additional file 4: Fig. S3a) or the mAARC ACLF score (Additional file 4: Fig. S3b).

## Discussion

The present study demonstrated that NLR had excellent performance for predicting short-term mortality risk in patients with HBV-ACLF; the prognostic value of NLR was superior to that of lactate, not only in the cirrhotic patient subgroup but also in the non-cirrhotic subgroup. NLR, a frequently-used and dependable inflammatory index, can be incorporated into the AARC ACLF scoring system for improving its prognostic accuracy, and benefiting the stratification and therapeutic guidance in HBV-ACLF patients admitted to primary hospitals.

High-level SI is often observed in the development of ACLF, presenting as activation of Toll-like and Nod-like receptors in peripheral myeloid cells (e.g., monocytes, dendritic cells, and neutrophils), overproduction of pro-inflammatory (e.g., tumor necrosis factor α [TNFα], interleukin-6 [IL-6], and IL-8) and anti-inflammatory cytokines (e.g., IL-10), and activated oxidative burst and recruitment of neutrophils [29, 30]. As ACLF progresses, the innate and adaptive immune cells gradually develop into suppression or paralysis, presenting as the exhaustion of immune cells (e.g., CD4^+^ T lymphocytes and CD8^+^ T lymphocytes) or effectors (e.g., interferon-γ, HLA-DR, TNFα, IL-6, and IL-8) [30–32], up-regulated expression of immune inhibitory receptors (e.g., programmed death-1, programmed death-ligand 1, and MER tyrosine kinase) [31, 33], impaired phagocytosis of myeloid cells [34, 35], and expansion of myeloid-derived suppressor cells [33, 34, 36]. Although disparity exists in the etiologies and definitions of ACLF between different regions, numerous studies have demonstrated that BI contributes to the development and progression of ACLF caused by non-HBV [5, 7, 8] or HBV [10, 11] etiologies. NLR serves as a simple inflammatory biomarker for assessing BI or SI, and may reflect the immunologic balance between the circulating neutrophils and lymphocytes in ACLF patients.

In contrast, elevation of serum lactate is caused by metabolic dysfunction (e.g., tissue hypoperfusion and decreased lactate clearance), and is often found in critically ill patients in the intensive care setting [37] and in patients with decompensated cirrhosis [38]. However, most HBV-ACLF patients evaluated in this study, particularly those from the validation cohort, were treated in the general ward, and around 23% of them were non-cirrhotic. Our data showed that compared with NLR, lactate had a weaker correlation with existing severity scores, insignificant reflection of the BI status in non-cirrhotic patient subgroup, and less accurate assessment of short-term mortality risk in patient subgroups with a low or medium level of lactate.

Our present study showed that compared with the lactate-based AARC ACLF score, the NLR-based mAARC ACLF score was more sensitive in predicting short-term mortality risk in patients with HBV-ACLF in the early phase, and its predictive value was less affected by the cirrhosis status of a patient. Additionally, 84 of 590 (14.8%) transplant-free patients with HBV-ACLF did not have data of the baseline lactate and were excluded in the analysis in this study, and 65 of them (77.4%) were from the validation cohort. Using three grades of NLR (< 3.10, 3.10–4.78, and > 4.78) instead of the corresponding levels of lactate (< 1.5 mmol/L, 1.5–2.5 mmol/L, and > 2.5 mmol/L) is more suitable for physicians in primary hospitals in this region because the routine blood test is more frequently taken than the lactate test at these centres.

Although WBC is incorporated in the CLIF-C ACLF score and the NACSELD ACLF score, we did not use this inflammatory index because disparity in the baseline values of WBC exists between cirrhotic and non-cirrhotic patient subgroups, or among cirrhotic patients using different management strategies. Our data showed that although there was overall statistical difference in baseline WBC between HBV-ACLF patients with or without admission BI, WBC did not exceed the upper limit of normal in more than half of the cirrhotic subgroup with admission BI. Additionally, ROC analysis showed that the performance of WBC was not inferior to that of lactate, but was significantly inferior to that of NLR. Thus, compared with the above-mentioned two complex scores, the mAARC ACLF score is significantly easier to calculate, and better suits patients with HBV-ACLF in primary hospitals in this region.

This study had some limitations in addition to its retrospective design. First, patients with non-HBV ACLF (e.g., alcoholic ACLF) were excluded to avoid discrepancy in baseline NLR values, and this may limit the generality of the mAARC ACLF score. Predictive values of NLR and the mAARC score need further validation in non-HBV ACLF patient populations. Additionally, the episodes of BI may be not accurately categorized because numerous patients diagnosed with healthcare-associated BI had long-term utility of preventive antibiotics, particularly extra-broad spectrum antibiotics in the primary facilities. Although significantly increased values of NLR and severity scores were observed in a significant proportion of ACLF patients diagnosed with hospital-acquired BI after 48 h of admission, we did not include their dynamic course in analysis because of objective limitations (e.g., lack of lactate or other laboratory variables for calculation of complex scores and inconsistent intervals of blood samples at different enrolled centres). The predictive values of NLR and the mAARC ACLF score need to be further validated in future prospective research.

## Conclusion

Our present study aimed first to use an NLR-based AARC ACLF scoring system for short-term mortality prediction in patients with HBV-ACLF. The findings indicate that the mAARC ACLF score is reliable, time-saving, and suitable for physicians in primary hospitals. Its prognostic accuracy needs further validation in patients with HBV-ACLF in other regions and in patients with non-HBV related ACLF. We implemented a multi-centre prospective study to further validate its prognostic value.

## Supplementary Information

**Additional file 1**. Table S1–S3.

**Additional file 2**. **Fig. S1:** Prognostic performance of the accepted ACLF scores and the mAARC ACLFs was compared within the non-cirrhotic (n = 67) or cirrhotic subgroup (n = 223) in the derivation cohort, and was stratified for the area under the receiver operating curve (AUROC) comparisons of 28-day mortality risk using (a); for AUROC comparisons of 90-day mortality risk using (b). The Hanley & McNeil method was used for AUROC comparisons. Abbreviations: ACLF, acute-on-chronic liver failure; CTP, Child-Turcotte-Pugh; MELD, model of end-stage liver disease; AARC ACLFs, Asian Pacific Society for the Study of the Liver ACLF research consortium ACLF score; mAARC ACLFs, modified AARC ACLFs.

**Additional file 3**. **Fig. S2:** Performance of NLR and lactate in predicting short-term mortality risk was compared in 204 patients with HBV-ACLF in the validation cohort, and was stratified for the area under the receiver operating curve (AUROC) comparisons for 28-day and 90-day mortality using (a) and (b); for the performance of lactate and NLR in the Kaplan-Meier (K-M) curve analysis of 90-day mortality using (c) and (d). Lactate was graded into three risk levels (<1.5 mmol/L, 1.5–2.5 mmol/L, and >2.5 mmol/L) according to the AARC ACLF score. NLR was divided into three risk levels (<3.10, 3.10–4.78, and >4.78) based on the upper limit of normal and the optimal cut-off value evaluated in the sensitivity analyses in the derivation cohort. The Hanley & McNeil method was used for AUROC comparisons. Abbreviations: NLR, neutrophil-to-lymphocyte ratio; ACLF, acute-on-chronic liver failure; HBV-ACLF, hepatitis b related ACLF; AARC, Asian Pacific Society for the Study of the Liver ACLF research consortium.

**Additional file 4**. **Fig. S3:** Prognostic performance of the AARC ACLFs and the mAARC ACLFs was compared using Kaplan-Meier (K-M) analysis in 204 patients with HBV-ACLF in the validation cohort, and was stratified for the performance of the AARC ACLFs using (a); for the performance of the mAARC ACLFs using (b). The mAARC ACLF score was calculated using three risk levels of baseline NLR (<3.10, 3.10–4.78, and >4.78) instead of corresponding levels of lactate (<1.5 mmol/L, 1.5–2.5 mmol/L, and >2.5 mmol/L). Abbreviations: ACLF, acute-on-chronic liver failure; HBV-ACLF, hepatitis b related ACLF; AARC ACLFs, Asian Pacific Society for the Study of the Liver ACLF research consortium ACLF score; mAARC ACLFs, modified AARC ACLFs.

## Data Availability

The datasets used and/or analysed during the current study are available from the corresponding authors on reasonable request.

## Abbreviations

ACLF: Acute-on-chronic liver failure
BI: Bacterial infection
SI: Systemic inflammation
WBC: White blood cell count
CLIF-C: Chronic Liver Failure Consortium
NACSELD: North American Consortium for the Study of End-Stage Liver Disease
HBV: Hepatitis b virus
HE: Hepatic encephalopathy
INR: International normalized ratio
APASL: Asian Pacific Society for the Study of the Liver
AARC: APASL ACLF research consortium
HBV-ACLF: HBV-related ACLF
NLR: Neutrophil-to-lymphocyte ratio
CTP: Child-Turcotte-Pugh
MELD: Model of end-stage liver disease
IQR: Interquartile range
AUROC: Area under the receiver operating characteristic curves
HBeAg: Hepatitis b envelope antigen
SBP: Spontaneous bacterial peritonitis
LA: Liver cirrhosis
mAARC ACLFs: Modified AARC ACLF score
TNF: Tumor necrosis factor
IL: Interleukin
