# Supplementary material for: A neutrophil-to-lymphocyte ratio-based prognostic model to predict mortality in patients with HBV-related acute-on-chronic liver failure
Source: BMC Gastroenterol. 2021 Nov 10;21:422. doi: 10.1186/s12876-021-02007-w (PMC8579631; doi:10.1186/s12876-021-02007-w)
Supplement: Supplementary file 1 — Additional file 1. Table S1–S3. [file 12876_2021_2007_MOESM1_ESM.docx]

**Supplementary Table 1** Baseline characteristics of survivors (n=164) vs non-survivors (n=126) in the derivation cohort

| Variable | Survivors  (n=164) | Non-survivors  (n=126) | *P* value |
| --- | --- | --- | --- |
| **Age (years)** | **43.1 ± 12.1** | **49 ± 11.4** | **<0.001** |
| Male,% (n/N) | 86.6 (142/164) | 87.3 (110/126) | 0.858 |
| HBeAg postive,% (n/N) | 48.2 (79/164) | 39.7 (50/126) | 0.149 |
| HBV DNA,% (n/N) |  |  |  |
| ≤200 IU/ml | 36.7 (56/164) | 35.9 (46/126) | 0.136 |
| 200–2×10 ^4^ IU/ml | 35.6 (59/164) | 35.9 (55/126) |  |
| ≥2×10 ^4^ IU/ml | 27.8 (49/164) | 28.1 (25/126) |  |
| Cirrhosis,% (n/N) | 70.7 (116/164) | 84.9 (107/126) | 0.004 |
| Ascites,% (n/N) | 50.0 (82/164) | 84.9 (107/126) | <0.001 |
| Bacterial infection,% (n/N) | 15.2 (25/164) | 77.8 (98/126) | <0.001 |
| Laboratory test |  |  |  |
| WBC (10^9^ /L) | 5.7 (4.3–7.7) | 9.2 (6.2–11.7) | <0.001 |
| NLR | 2.8 (2.0–4.3) | 7.9 (5.4–10.7) | <0.001 |
| Platelets (10^9^ /L) | 97 (62–137) | 85 (50–112) | 0.013 |
| **TB (mg/dL)** | **17.3 ± 8.9** | **24.7 ± 11.5** | **<0.001** |
| **Albumin (g/L)** | **33.3 ± 4.8** | **32.2 ± 5.5** | **0.032** |
| Creatinine (µmol/L) | 63 (55–73) | 70 (57–98) | 0.005 |
| Sodium (mmol/L) | 137 (135–140) | 134 (130–138) | <0.001 |
| Glucose (mmol/L) | 5.5 (4.3–7.3) | 6.1 (4.6–8.3) | 0.053 |
| Lactate (mmol/L) | 1.7 (1.2–2.3) | 2.3 (1.4–3.7) | <0.001 |
| INR | 1.8 (1.5–2.2) | 2.6 (2.1–3.6) | <0.001 |
| HE grade | 0 (0–0) | 1 (1–2) | <0.001 |
| Severity score |  |  |  |
| **CTP score** | **10 (9–11)** | **12 (11–13)** | **<0.001** |
| **MELD score** | **20.6 ± 5.4** | **28.9 ± 9.0** | **<0.001** |
| **MELD-Na score** | **21 (17–25)** | **30.5 (24–40)** | **<0.001** |
| AARC ACLF score | 9 (7–8) | 11 (9–12) | <0.001 |
| 5–7 | 38.4 (63/164) | 5.6 (7/126) | <0.001 |
| 8–10 | 55.5 (91/164) | 38.1 (48/126) |  |
| 11–15 | 6.1 (10/164) | 56.3 (71/126) |  |
| Antiviral history,% (n/N) |  |  |  |
| Naïve | 70.7 (116/164) | 67.5 (85/126) | 0.549 |
| Non–naïve | 29.3 (48/164) | 32.5 (41/126) | 0.549 |
| Poor adherence | 70.8 (34/48) | 87.8 (36/41) | 0.051 |
| Antibiotic history,% (n/N) | 29.3 (48/164) | 56.4 (71/126) | <0.001 |
| **Artificial liver support history**  **% (n/N)** | 10.4 (17/164) | 34.9 (44/126) | <0.001 |

**Continuous variables with normal distributions were described using mean ± SD. Continuous variables with skewed distributions were described using median with interquartile range (IQR) and compared using the Mann‐Whitney U test.** Categorical variables were expressed as percentages (frequencies) and compared using the Chi‐square test followed by Fisher’s exact test, as appropriate.

Abbreviations: HBeAg, hepatitis b envelope antigen; HBV, hepatitis b virus; WBC, white blood cell count; NLR, neutrophil/lymphocyte ratio; TB, total bilirubin; INR, international normalized ratio; HE, hepatic encephalopathy; CTP, Child-Turcotte-Pugh; MELD, model for end-stage liver disease; ACLF, acute-on-chronic liver failure; AARC, Asian Pacific Society for the Study of the Liver ACLF research consortium.

**Supplementary Table 2** Episodes of admission bacterial infection in the derivation and validation cohorts

| Variable [% (n/N)] | Total  (n=494) | Derivation  cohort (n=290) | Validation  cohort(n=204) | *P* value |
| --- | --- | --- | --- | --- |
| Catergories of acquisition |  |  |  |  |
| Overall | 38.5 (190/494) | 42.4 (123/290) | 32.8 (67/204) | 0.031 |
| Community-acquired | 27.4 (52/494) | 20.3 (25/123) | 40.3 (27/67) | 0.003 |
| Healthcare-associated | 72.6 (138/494) | 79.7 (98/123) | 59.7 (40/67) | 0.003 |
| Sites of infection |  |  |  |  |
| Pneumonia | 20.6 (102/494) | 26.2 (76/290) | 12.7 (26/204) | <0.001 |
| Spontaneous bacterial peritonitis | 16.2 (80/494) | 17.9 (52/290) | 13.7 (28/204) | 0.212 |
| Biliary tract infection | 4.0 (20/494) | 3.8 (11/290) | 4.4 (9/204) | 0.731 |
| Others ^†^ | 4.0 (20/494) | 5.2 (15/290) | 2.5 (5/204) | 0.131 |
| Unproven sites ^‡^ | 2.6 (13/494) | 1.0 (3/290) | 4.9 (10/204) | 0.007 |
| Microbiological evidence | 12.3 (61/494) | 11.4 (33/290) | 13.7 (28/204) | 0.435 |
| Multidrug-resistance | 29.5 (18/61) | 33.3 (11/33) | 25.0 (7/28) | 0.477 |

Categorical data are expressed as percentages (frequencies) and analysed with the Chi‐square test followed by Fisher’s exact test, as appropriate.

^†^ included sepsis (n = 6), urinary tract infection (n = 4), intestinal infection (n = 4), soft tissue infection (n = 3), meningitis (n = 2), and spleen abscess (n = 1).

^‡^ Patients who had the presence of fever and leukocytosis and required antibiotic therapy without any identifiable source.

**Supplementary Table 3** Baseline characteristics of survivors (n = 128) vs non-survivors (n = 76) in the validation cohort

| Variable | Survivor  (n=128) | Non-survivor  (n=76) | *P* value |
| --- | --- | --- | --- |
| Age (years) | **44.3 ± 13.5** | **52 ± 10.4** | **<0.001** |
| Male,% (n/N) | 84.4 (108/128) | 80.3 (61/76) | 0.451 |
| HBeAg postive,% (n/N) | 43.0 (55/128) | 39.5 (30/76) | 0.625 |
| HBV DNA,% (n/N) |  |  |  |
| ≤200 IU/ml | 16.8 (22/128) | 19.1 (24/76) | 0.011 |
| 200–2×10 ^4^ IU/ml | 24.2 (31/128) | 18.0 (23/76) |  |
| ≥2×10 ^4^ IU/ml | 58.9 (75/128) | 22.3 (29/76) |  |
| Cirrhosis,% (n/N) | 69.5 (89/128) | 90.8 (69/76) | <0.001 |
| Ascites,% (n/N) | 55.5 (71/128) | 88.2 (67/76) | <0.001 |
| Bacterial infection,% (n/N) | 11.7 (15/128) | 68.4 (52/76) | <0.001 |
| Laboratory test |  |  |  |
| WBC (10^9^ /L) | 5.4 (4.2–6.8) | 7.8 (5.2–11.0) | <0.001 |
| NLR | 2.7 (1.8–4.2) | 8.3 (5.6–11.8) | <0.001 |
| Platelets (10^9^ /L) | 95 (69–132) | 70 (42–125) | 0.003 |
| **TB (umol/L)** | **16.8 ± 8.7** | **22.5 ± 11.3** | **<0.001** |
| **Albumin (g/L)** | **32.1 ± 5.0** | **31.0 ± 6.0** | **0.142** |
| **Creatinine (µmol/L)** | **64.9 ± 16.1** | **108.5 ± 104.3** | **0.003** |
| Sodium (mmol/L) | 138 (135–140) | 135 (130–138) | <0.001 |
| Glucose (mmol/L) | 5.3 (4.1–8.3) | 7.3 (4.5–9.5) | 0.071 |
| Lactate (mmol/L) | 1.6 (1.2–2.2) | 1.9 (1.4–3.0) | 0.008 |
| INR | 1.6 (1.5–2.0) | 2.2 (1.9–3.0) | <0.001 |
| HE grade | 0 (0–0) | 1 (1–2) | <0.001 |
| Severity score |  |  |  |
| **CTP score** | **10 (9–11)** | **12 (11–13)** | **<0.001** |
| MELD score | 20 (17–22) | 26 (22–31) | <0.001 |
| **MELD-Na score** | **20 (17–23)** | **28 (23–39)** | **<0.001** |
| AARC ACLF score | 7 (6–9) | 10 (9–11) | <0.001 |
| 5–7 | 53.1 (68/128) | 5.3 (4/76) | <0.001 |
| 8–10 | 42.2 (54/128) | 51.3 (39/76) |  |
| 11–15 | 4.7 (6/128) | 43.4 (33/76) |  |
| Antiviral history,% (n/N) |  |  |  |
| Naïve | 77.3 (99/128) | 68.4 (52/76) | 0.160 |
| Non-naïve | 22.7 (29/128) | 31.6 (24/76) | 0.160 |
| Poor adherence | 69.0 (20/29) | 79.2 (19/24) | 0.838 |
| Antibiotic history,% (n/N) | 16.4 (21/128) | 52.6 (40/76) | <0.001 |
| **Artificial liver support history**  **% (n/N)** | 8.6 (11/128) | 30.3 (23/76) | <0.001 |

**Continuous variables with normal distributions were described using mean ± SD. Continuous variables with skewed distributions were described using median with interquartile range (IQR) and compared using the Mann‐Whitney U test.** Categorical variables were expressed as percentages (frequencies) and compared using the Chi‐square test followed by Fisher’s exact test, as appropriate.

Abbreviations: HBeAg, hepatitis b envelope antigen; HBV, hepatitis b virus; WBC, white blood cell count; NLR, neutrophil/lymphocyte ratio; TB, total bilirubin; INR, international normalized ratio; HE, hepatic encephalopathy; CTP, Child-Turcotte-Pugh; MELD, model for end-stage liver disease; ACLF, acute-on-chronic liver failure; AARC, Asian Pacific Society for the Study of the Liver ACLF research consortium.
