# Supplementary figures and images for: A neutrophil-to-lymphocyte ratio-based prognostic model to predict mortality in patients with HBV-related acute-on-chronic liver failure
Source: BMC Gastroenterol. 2021 Nov 10;21:422. doi: 10.1186/s12876-021-02007-w (PMC8579631; doi:10.1186/s12876-021-02007-w)

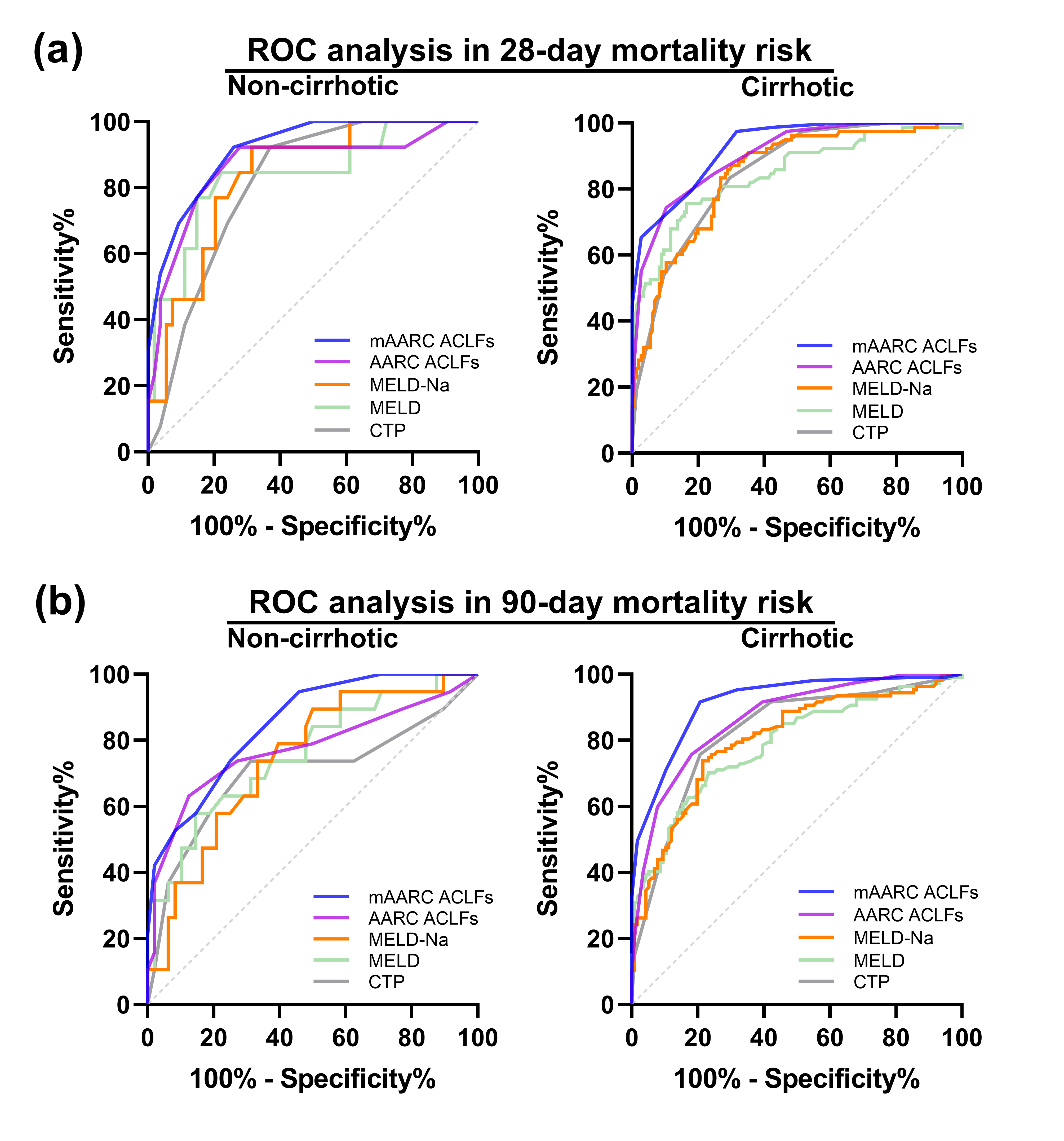

Supplement: Supplementary file 2 — Additional file 2. Fig. S1: Prognostic performance of the accepted ACLF scores and the mAARC ACLFs was compared within the non-cirrhotic (n = 67) or cirrhotic subgroup (n = 223) in the derivation cohort, and was stratified for the area under the receiver operating curve (AUROC) comparisons of 28-day mortality risk using (a); for AUROC comparisons of 90-day mortality risk using (b). The Hanley & McNeil method was used for AUROC comparisons. Abbreviations: ACLF, acute-on-chronic liver failure; CTP, Child-Turcotte-Pugh; MELD, model of end-stage liver disease; AARC ACLFs, Asian Pacific Society for the Study of the Liver ACLF research consortium ACLF score; mAARC ACLFs, modified AARC ACLFs. [file 12876_2021_2007_MOESM2_ESM.tif]

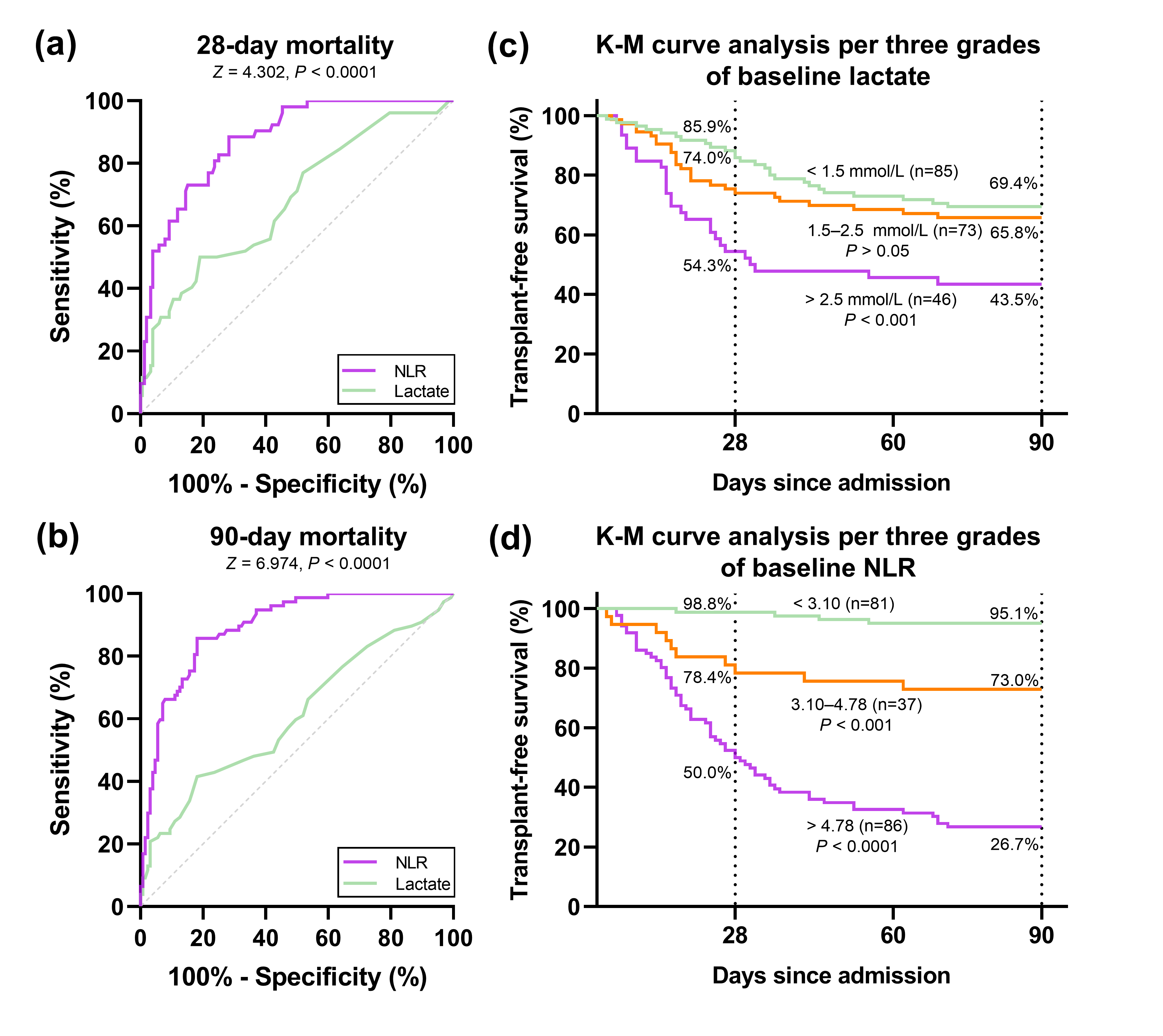

Supplement: Supplementary file 3 — Additional file 3. Fig. S2: Performance of NLR and lactate in predicting short-term mortality risk was compared in 204 patients with HBV-ACLF in the validation cohort, and was stratified for the area under the receiver operating curve (AUROC) comparisons for 28-day and 90-day mortality using (a) and (b); for the performance of lactate and NLR in the Kaplan-Meier (K-M) curve analysis of 90-day mortality using (c) and (d). Lactate was graded into three risk levels (<1.5 mmol/L, 1.5–2.5 mmol/L, and >2.5 mmol/L) according to the AARC ACLF score. NLR was divided into three risk levels (<3.10, 3.10–4.78, and >4.78) based on the upper limit of normal and the optimal cut-off value evaluated in the sensitivity analyses in the derivation cohort. The Hanley & McNeil method was used for AUROC comparisons. Abbreviations: NLR, neutrophil-to-lymphocyte ratio; ACLF, acute-on-chronic liver failure; HBV-ACLF, hepatitis b related ACLF; AARC, Asian Pacific Society for the Study of the Liver ACLF research consortium. [file 12876_2021_2007_MOESM3_ESM.tif]

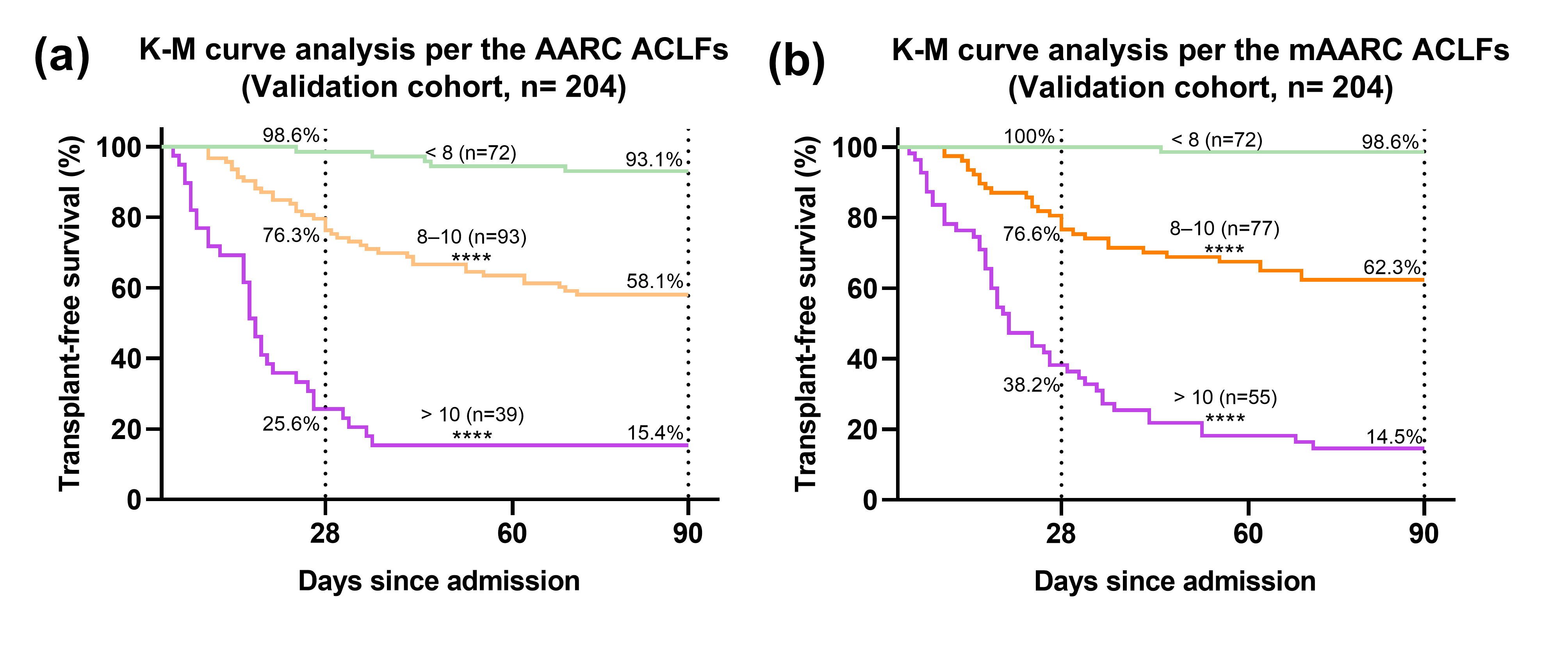

Supplement: Supplementary file 4 — Additional file 4. Fig. S3: Prognostic performance of the AARC ACLFs and the mAARC ACLFs was compared using Kaplan-Meier (K-M) analysis in 204 patients with HBV-ACLF in the validation cohort, and was stratified for the performance of the AARC ACLFs using (a); for the performance of the mAARC ACLFs using (b). The mAARC ACLF score was calculated using three risk levels of baseline NLR (<3.10, 3.10–4.78, and >4.78) instead of corresponding levels of lactate (<1.5 mmol/L, 1.5–2.5 mmol/L, and >2.5 mmol/L). Abbreviations: ACLF, acute-on-chronic liver failure; HBV-ACLF, hepatitis b related ACLF; AARC ACLFs, Asian Pacific Society for the Study of the Liver ACLF research consortium ACLF score; mAARC ACLFs, modified AARC ACLFs. [file 12876_2021_2007_MOESM4_ESM.tif]
